# Long-Lasting Sex-Specific Effects Based On Emotion- and Cognition-Related Behavioral Assessment of Adult Rats After Post-Traumatic Stress Disorder From Different Lengths of Maternal Separation

**DOI:** 10.3389/fpsyt.2019.00289

**Published:** 2019-06-04

**Authors:** Rucui Yang, Haoran Sun, Yani Wu, Guohua Lu, Yanyu Wang, Qi Li, Jin Zhou, Hongwei Sun, Lin Sun

**Affiliations:** ^1^Department of Clinical Medicine, Weifang Medical University, Weifang, China; ^2^Department of Psychology, Weifang Medical University, Weifang, China; ^3^Department of Psychiatry and Centre for Reproduction Growth and Development, University of Hong Kong, Hong Kong, Hong Kong; ^4^College of Pharmacy, Weifang Medical University, Weifang, China

**Keywords:** maternal separation (MS), early-life stress, post-traumatic stress disorder (PTSD), single prolonged stress (SPS), gender difference, behavioral test

## Abstract

Adverse early life stress is a major cause of vulnerability to various mental disorders in adulthood, including post-traumatic stress disorder (PTSD). Recent studies have suggested that early life stress can help the body adapt optimally when faced with stressful trauma in adult life. An interaction may exist between early life stress (e.g., childhood trauma) and vulnerability to PTSD. This study aimed to evaluate emotion-related behaviors and verify the long-lasting effects of cognitive aspects of PTSD after exposure to severe adverse early life stress, such as long-term separation. Adverse early life stress was simulated by subjecting rats to 3 or 6 consecutive hours of maternal separation (MS) daily, from postnatal day (PND) 2 to PND 14. Single-prolonged stress (SPS) was simulated on PND 80 to imitate other adulthood stresses of PTSD with gender divisions (M-MS3h-PTSD, F-MS3h-PTSD, M-MS6h-PTSD, F-MS6h-PTSD, M-PTSD, and F-PTSD). After the MS and PTSD sessions, behavioral tests were conducted to assess the effectiveness of these treatments, which included an open field test (OFT), elevated plus maze test (EPMT), water maze test (WMT), and forced swimming test (FST) to detect anxiety-like behavior (OFT and EPMT), memory behavior (WMT), and depressive behavior (FST). The M-MS3h-PTSD group had fewer time entries into the open arms of EPMT than the F-MS3h-PTSD group, and the M-MS6h-PTSD group demonstrated fewer up-right postures in the OFT than the F-MS6h-PTSD group. The M-MS3h-PTSD group exhibited more exploratory behavior than the M-MS6h-PTSD and M-PTSD groups in the OFT. Less exploratory behavior was observed in the F-MS3h-PTSD group than in the F-MS6h-PTSD group, which demonstrated significantly increased freezing times in the FST compared to the F-PTSD group. The WMT revealed significant differences in learning and memory performance between the M-MS3h-PTSD group and other treatment groups, which were not found in the female rats. These findings demonstrate that an early stressful experience, such as MS, may be involved in helping the body adapt optimally when faced with additional trauma in adulthood, although mild early life stress might benefit learning and memory among males.

## Introduction

Studies have found that regions of the brain that are especially susceptible to stress during the first 7 years of life (1–3) are involved in detecting and responding to threats and in regulating stress responses. Modifying this system is one of the primary ways human brains are shaped by early adversity (4). For instance, studies on children raised in orphanages—where they lack proper social and maternal support—have reported lasting adverse effects on cognitive functioning and increased risk for psychiatric disorders (5, 6). Epidemiological studies have indicated that adverse early life events can increase one’s risk of developing psychopathology in adulthood (7). Although child abuse is a serious social problem in most countries, an understanding of various types of separations and corresponding gender differences in adulthood remains unclear.

Early postnatal stress animal models have been developed due to the high prevalence and serious consequences of early stress in humans. Rats exposed to early-life stresses, such as maternal separation (MS) during lactation, serve as good models for studying the effects that neglect has in early life (8). The use of animal models to induce early life stress is common when studying long-term consequences of stress (9). Researchers have performed MS in various ways, such as different lengths of separation; some authors separated animals for 3h (10) every day, whereas others separated them from 4h (11) or 6h (12). Only a few papers have compared different lengths of MS (13, 14).

Post-traumatic stress disorder (PTSD) is a highly disabling condition observed in individuals following exposure to severe emotional or physically life-threatening traumatic events (12), compounded by genetic and environmental factors (15). Early life stress and its associated changes in several hormonal states induce structural alterations. These alterations then induce disorders of brain functions throughout life (16). A focus on reliance has been largely ignored in relevant genetic and environmental interaction studies, referring to a dynamic pattern of positive adaptation despite experiencing significant trauma or adversity (17). Early environmental events exert enduring effects on behavioral parameters related to coping with stress, and animal models of stress represent an invaluable tool for investigating the complex relationship between brain development and stress (18, 19). In this study, the MS model was used to mimic early life stress and a potential influencing factor of PTSD.

Repeated MS is thought to increase animals’ sensitivity to stress (9). Some studies have confirmed that the interaction of perinatal exposure to adversity with individual genetic liabilities may increase an individual’s vulnerability to psycho- and physiopathology throughout life (20). However, other studies have cited data supplementing other reports, questioning whether early life stress is always detrimental later in life (21–23). Adolescence is a life period during which behavioral development is particularly susceptible to social influences. Stressful social events during this time have been found to alter and canalize behavior in an adaptive fashion or enhance specific types of cognitive performance, such that earlier influences on behavioral development are complemented and modified (23, 24). Macrì and Würbel proposed that perinatal low-to-moderate stressful situations could induce “protective” effects in adulthood; however, severely stressful situations can result in detrimental effects. Although several studies have demonstrated that as a case of early handling, brief daily separation from the mother during the neonatal phase can attenuate the effects of chronic stress on inducing hypothalamic-pituitary-adrenal (HPA) axis reactivity (25), these effects in animal models of PTSD have not been examined.

The aim of the present study was to compare the functional consequences of the PTSD model after MS at either 3h or 6h every day over a wide range of behaviors, which were either emotion-related (anxiety and depression) or learning-related (e.g., memory) in adulthood. The PTSD model after MS in rats imitates orphans or left-behind children exposed to a traumatic event (or events), as it alters vulnerability to psychiatric disorders and cognitive deficits in adult life. The sex of rats was considered because sexual differences in enduring early-life stress could induce rodents’ alterations in neurogenesis (26).

## Materials and Methods

### Animals

Pregnant Sprague–Dawley (SD) rats were bred at our animal facility and randomly selected from the Animal Center of Weifang Medical University. The day of birth was recorded as postnatal day (PND) 0. Pups were subjected to either MS or animal-facility-reared treatment (27). Rats were divided into 4 groups: control, no MS, MS 3h, and MS 6h per day from PND 2 to PND 14. After that, the pups were weaned and kept with rats of the same sex, from which 7 male pups and 7 female pups were randomly selected from the groups that we mentioned before. The rest were removed to ensure an equal number of males and females per litter. The 56 animals (220–350g at the end of the experiment) were housed in a limited-access rat facility with 7 rats per polycarbonate cage at a constant room temperature (22 +/−2°C), humidity (55 ± 15%), and an artificial light-dark cycle of 12h (08:00–20:00/20:00–08:00). We performed model-building tasks at 13:00 and 16:00, after animals had reached adulthood. These experiments were designed to minimize the number of the animals used and were conducted in line with National Institutes of Health Guidelines (Use of Laboratory Animals) and ethical standards, including ethics committee approval as well as consent procedure. Experiments were approved by the Animal Care and Use Committee of Weifang Medical University.

### Maternal Separation

Litters were randomly assigned to be reared under animal-facility-rearing conditions or undergo MS. Animals were randomly divided into 8 groups and their behavioral parameters were recorded separately: male control group (M-Control, *n* = 7), male rats with PTSD (M-PTSD, *n* = 7), male rats with 3h MS and PTSD (M-MS3h-PTSD, *n* = 7), male rats with 6h MS and PTSD (M-MS6h-PTSD, *n* = 7), female control group (F-Control, *n* = 7), female rats with PTSD (F-PTSD, *n* = 7), female rats with 3h MS and PTSD (F-MS3h-PTSD, *n* = 7), and female rats with 6h MS and PTSD (F-MS6h-PTSD, *n* = 7). In the F/M-MS3h-PTSD groups, litters were separated from their dams for 3h (9:00–12:00) each day (10, 19). In the F/M-MS6h-PTSD groups, litters were separated from their dams for 6h (9:00–15:00) each day (PND 2-PND 14). During separation, each dam was removed from its maternity cage and placed into an identical cage until the end of the separation period. The pups were then removed from the nest, placed in an incubator (30°C with humidity 55 ± 15%), and transferred to an adjacent room to eliminate influences of the dams’ odor and sounds. At the end of the separation period, pups were returned to their maternity cages and reunited with the dams. Pups without MS in the F/M-PTSD groups were left with the dams.

### The Establishment of PTSD Model

Single prolonged stress (SPS) procedures were similar to previous experiments (28), conducted in three stages. Animals were immobilized for 2h (IMO 2h). Rats were then immediately subjected to a 20-min forced swim (FS 20min). After removal, they were dried and allowed 15 min to recover (Rest 15min) under a heating lamp before being exposed to diethyl ether until loss of consciousness. The SPS procedure refers to the application of the three stressors and a 7-day quiescent period (28). The quiescent period is critical for developing behavioral and PTSD-like physiological abnormalities after SPS (29). Behavioral experiments were generally performed approximately 7 days after establishing the PTSD model.

## Experimental Procedures

**Figure 1:**
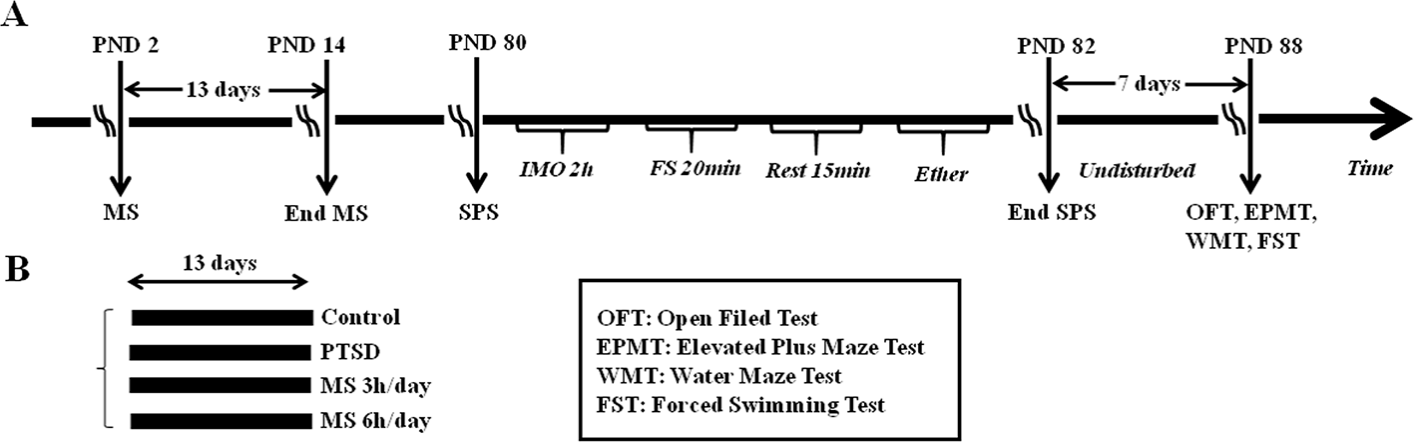
**(A)** The process diagram of the experiment. SPS was conducted in three stages. Rats were immobilized for 2h (IMO 2 h), forced to swim for 20 min (FS 20 min), allowed to recover with a heating lamp for 15 min, and then exposed to diethyl ether until loss of consciousness (Ether). Rats were then returned to their home cages and left undisturbed for 7 days until the experimental manipulations. **(B)** The experiment included the control group (*n* = 14), PTSD group without MS (*n* = 14), PTSD-treated with prior 3h MS (*n* = 14), PTSD-treated with prior 6h MS (*n* = 14) or simply ‘Control,’ ‘PTSD,’ ‘MS3h-PTSD’ and ‘MS6h-PTSD’, respectively. Behavior experiments included OFT, EPMT, WMT and FST.

All animals underwent all behavioral experiments, which were conducted sequentially with the same sequences: 1) open field test (OFT), 2) elevated plus maze test (EPMT), 3) water maze test (WMT), and 4) forced swimming test (FST) (**Figure 1**). This sequence was selected based on the principle of preference for light-pressure and general requirements for the operation of animal experiments (30, 31).

### Anxiety-Like Behavior

**Open field test.** The chamber was a box without a lid (100 × 100 × 50 cm) consisting of a black floor and walls. Incandescent bulbs installed on the ceiling were used for illuminating the box. Each rat was transported from the colony room to the OFT room on the day of OFT. At the beginning of the experiment, the animals were placed gently in the corner and allowed to explore the chamber freely for 5 min, and upright numbers were recorded at the same time. Automated image analysis software (SMART 3.0, Panlab SL; Barcelona, Spain) was used for recording time spent in central areas, which evaluated the anxiety-like behavior. The open field was cleaned with ethanol after each test.

**Elevated plus maze test.** The EPMT is commonly used to score PTSD-like anxiogenic behavior in rats by exploiting the conflict between rats’ innate fear of open spaces versus their desire to explore novel environments (32). The elevated plus maze was 50 cm above the floor and consisted of two open arms (50 cm × 10 cm) and two enclosed arms (50 cm × 10 cm × 40 cm) with an open roof extending from a center platform (10 cm × 10 cm). The structures were arranged so the two open arms were opposite each other. All the rats were placed gently in the central platform, facing the closed arm and allowed to explore the area freely for 5 min. After each test the maze was wiped with ethanol. A video camera was positioned above the apparatus to record each animal’s behavior, namely the following parameters: the number of open arm entries and the time spent in the open arms. An entry was counted when a rat’s four paws entered a closed or open arm. The shorter the time spent in open arms, the higher the rat’s anxiety.

### Learning and Memory Behavior

**Water maze test.** The WMT can be used for measuring spatial navigation learning and memory in rats (33). We utilized a 160-cm-diameter pool filled with water to a depth of 30 cm and maintained at 23 ± 1°C. The pool had a 20×20 cm escape platform, which was hidden 1 cm below the water surface in the center of one quadrant of the pool. The platform was in the same location during the training session, and the order of start locations was quasi-random. Four prominent shapes were placed on the poor’s wall (one per quadrant: square, heart, triangle and moon), and the water was changed daily. Rats were placed in the pool and allowed to search for the platform for 90 s. The rat was guided to the platform and allowed to remain there for 20 s to recognize the location if it did not find the platform after 90 s. Rats were subjected to four sequential trials each day at an interval of 30 s, during which the rat was placed in its home cage in a different room. The training session lasted 4 days. The probe trial was performed on day 5, when the platform was removed. The escape latency time required for the rat to find and climb onto the platform was recorded. Each training session was recorded by a video camera mounted above the center of the tank, and movement data was analyzed using SMART 3.0.

### Depressive Behavior

**Forced swimming test.** The FST is a test used for measuring depression-like behavior in rodents (31, 34). On the first day, we placed rats individually into a cylindrical tank composed of a transparent Plexiglas cylinder (diameter: 20 cm; height: 46 cm) to a depth of 36 cm filled with warm water (23–24°C) for 15 min (pretest session) in order to adapt to the apparatus better. At the same time on the following day, the rats were put into the cylinders for 5 min, after which they were removed from the cylinder, returned to their home cage and dried with a towel. Each rat assumed an immobile posture after being placed in the water, defined as the state in which rats made only the movements necessary to keep their head above the surface of water (35). The duration of each rat’s immobility at 5 min on the second day was scored using videotapes by a trained observer who was blind to the experimental conditions. Water in the tank was guaranteed to be changed after each trial. We referred to the process of Jong-Ho Lee et al., and Lanwei Hou et al. (11, 36) during this test.

An overview of the experimental procedure is displayed in **Figure 1**.

## Statistical Analysis

Data were expressed as mean ± standard error. We deleted the values with the largest difference in each group to ensure the credibility of the data, so the final quantity is 6. The OFT, EPMT, probe test of WMT, and FST results were analyzed using analysis of variance (ANOVA) with Tukey. Independent-samples t-test was used for the comparison of gender difference. A repeated-measures multivariate analysis of variance (MANOVA) was conducted to analyze of WMT escape latencies using SPSS statistical software (Version 22.0, SPSS, Inc.; Chicago, IL, USA) at a significance level of *p* < .05 and values are means ± standard error.

## Results

### Analysis of Anxiety-Like Behavior

**Figure 2:**
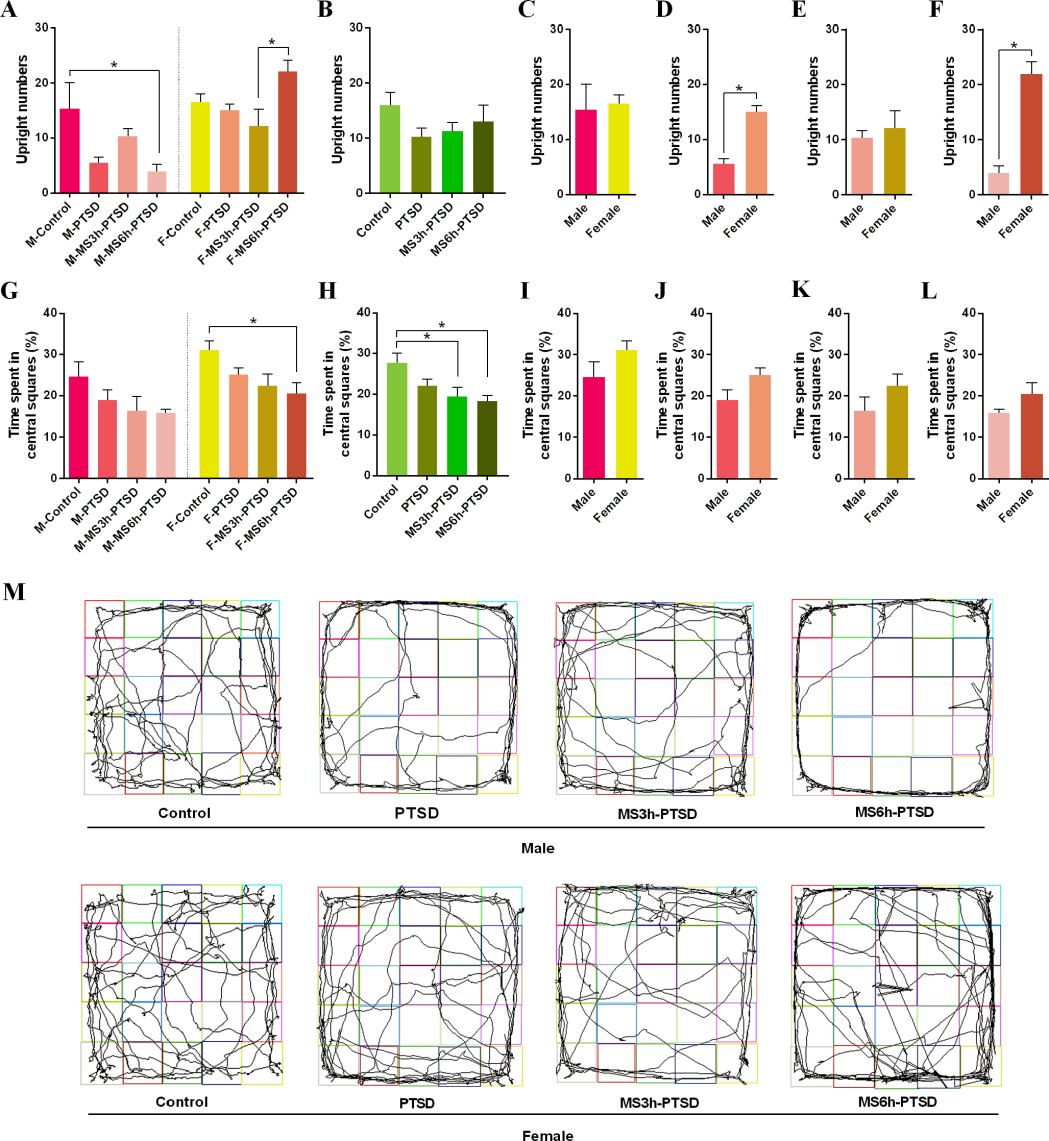
Anxiety-related effect that MS had from PND 2 to PND 14 after subjecting the animals to the SPS as a PTSD model process in adulthood. **(A)** and **(B)**: Upright numbers in the OFT. **(G)** and **(H)**: Time spent in central squares (%). **(A)** and **(G)**: Divided by gender and time difference of MS. **(B)** and **(H)**: Divided by time difference of MS. **(C)**, **(D)**, **(E)**, **(F)**, **(I)**, **(J)**, **(K)** and **(L)**: Gender difference. **(M)**: Representative video tracking images during 5 min in the OFT. Values are means ± standard error. *p < .05 was statistically significant.

The F-MS3h-PTSD group demonstrated less upright time than the F-MS6h-PTSD rats (*F*(3,20) = 3.88, *p* = .025; Post hoc test: *p* = .017). The M-MS3h-PTSD and F-MS3h-PTSD rats showed no obvious gender differences between upright times (**Figure 2A**). Rats in the F-PTSD and F-MS6h-PTSD group exhibited significantly more upright time in the OFT apparatus than the M-PTSD and M-MS6h-PTSD rats (*t* = 7.432, *p* < .001; *t* = 6.154, *p* < .001; **Figure 2D, F**). In other cases there was no gender difference (**Figure 2C, E, I, G, K and L**). The M-MS6h-PTSD group and F-MS6h-PTSD group had less upright numbers and time spent in the central squares compared with their control groups (*F*(3,20) = 3.97, *p* = .023, Post hoc test: *p* = .026; *F*(3,20) = 3.59, *p* = .032, Post hoc test: *p* = .028), (**Figure 2A, G**). The tracking images is shown in the figure (**Figure 2M**).

**Figure 3:**
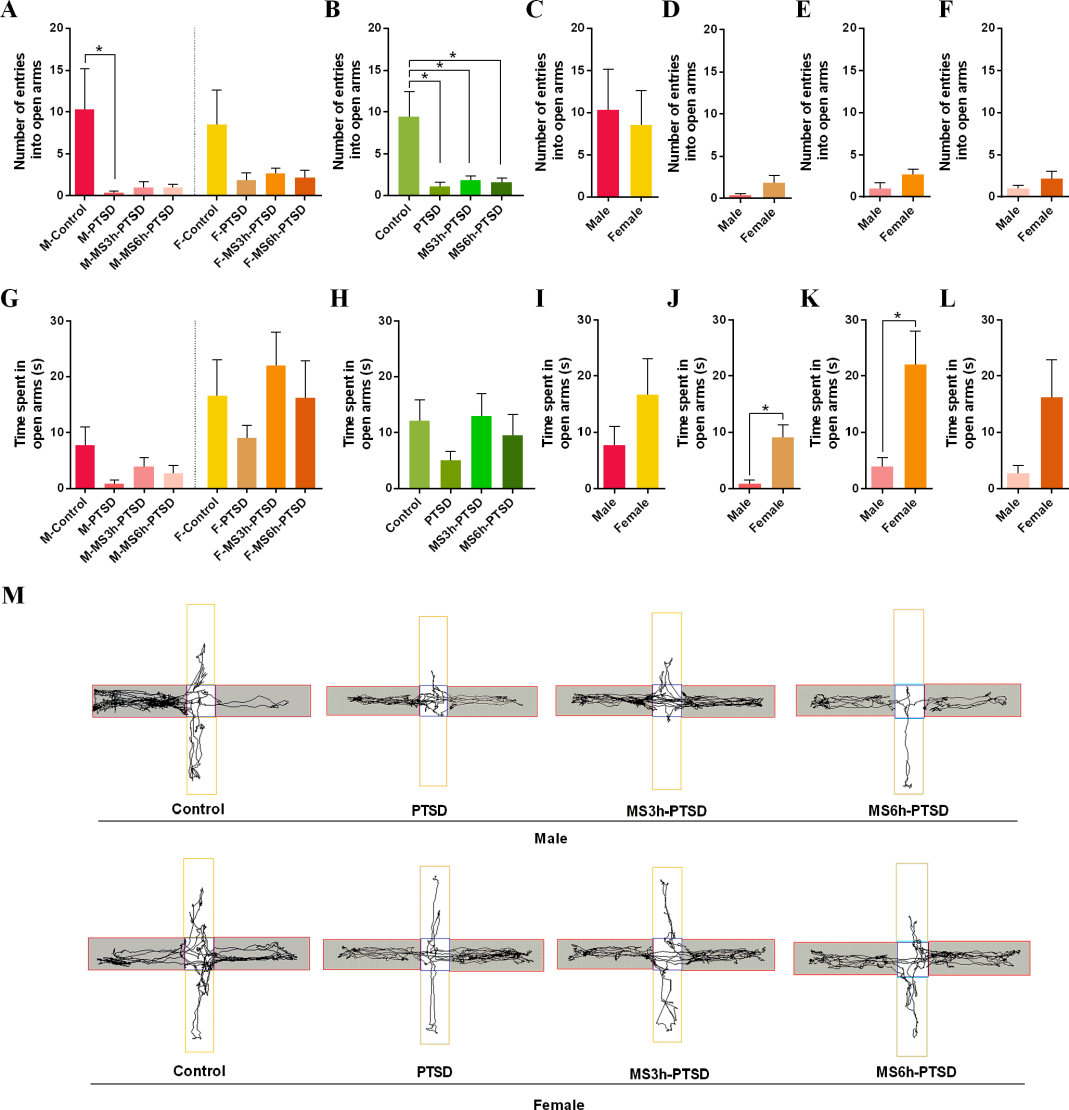
Anxiety-related effect that MS had from PND 2 to PND 14 after subjecting the animals to the SPS as a PTSD model process in adulthood. **(A)** and **(B)**: Number entering in open arms in the EPMT. **(G)** and **(H)**: Time spent in open arms (s) in the EPMT. **(A)** and **(G)**: Divided by gender and time difference of MS. **(B)** and **(H)**: Divided by time difference of MS. **(C)**, **(D)**, **(E)**, **(F)**, **(I)**, **(J)**, **(K)** and **(L)**: Gender difference. **(M)**: Representative video tracking images during 5 min in the EPMT. Values are means ± standard error. *p < .05 was statistically significant.

The numbers of entries into the open arms in EPMT were not significantly different between the M-MS3h-PTSD and M-MS6h-PTSD groups and the M-PTSD group on PND 88; the same results were found in the females. The M-PTSD rats had less time entering the open arms when compared to the control group; however, the female rats suggested no significant differences between entering the open arms or the opposite side (**Figure 3A**). The female rats had more upright postures of OFT and time entering the open arms of the EPMT than the male rats with MS and PTSD (**Figure 2F**, **Figure 3K**). The results of time spent in the open arms in the M-MS3h-PTSD and M-MS6h-PTSD groups were not significantly different from the M-PTSD group, and no difference was found between the F-MS3h-PTSD and F-MS6h-PTSD groups and the F-PTSD group (**Figure 3G**). Rats in the M-MS3h-PTSD group spent significantly less time in the open arms of the EPMT than the F-MS3h-PTSD group (*t* = 2.936, *p* = .015; see **Figure 3K**). In other cases there was no gender difference (**Figure 3C–F, I and L**). The tracking images was shown in the figure (**Figure 3M**).

No significant difference between the groups was found when there were no gender differences in upright numbers of OFT and time spent in open arms of EPMT (**Figure 2B**, **Figure 3G**). There were statistically significant differences between the control groups and the experimental groups in the time spent in central squares of OFT (MS3h-PTSD: *p* = .021; MS6h-PTSD: *p* = .007) and number of entries into open arms of EPMT (PTSD: *p* = .003; MS3h-PTSD: *p* = .008; MS6h-PTSD: *p* = .006), (**Figure 2H**, **Figure 3B**). When consider time and gender, no difference was found about time spent in open arms (**Figure 3H**). Detailed data information can be found in the **Supplementary Material**.

### Analysis of Learning and Memory Behavior

**Figure 4:**
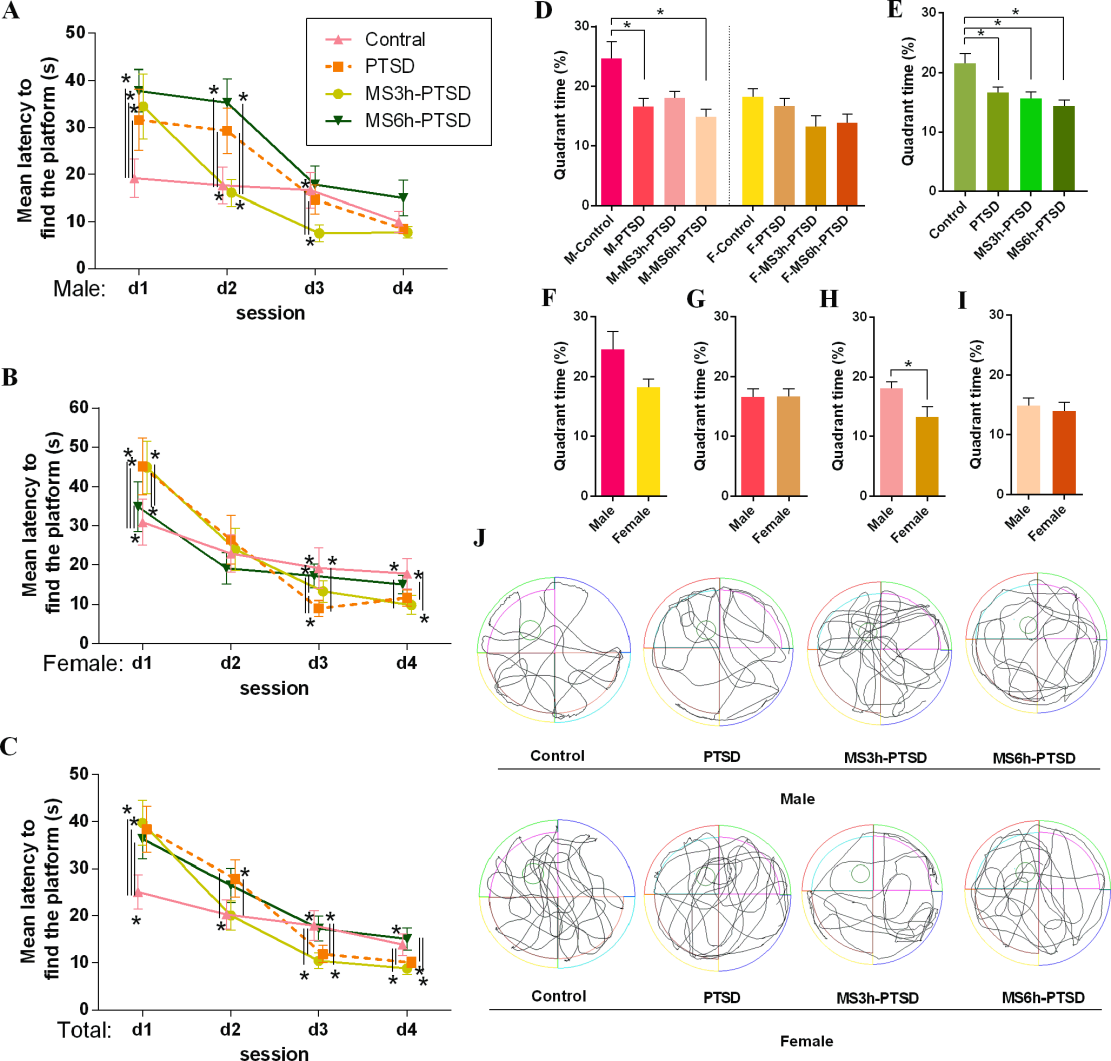
Learning and memory-related effect that MS had from PND 2 to PND 14 after subjecting the animals to the process of SPS as a PTSD model in adult age. **(A)**, **(B)** and **(C)**: Mean latency to find the platform (s) in the spatial learning test in the WMT. **(D)** and **(E)**: Quadrant time (%) of probe test in the WMT. **(D)**: Divided by gender and time difference of MS. **(E)**: Divided by time difference of MS. **(F)**, **(G)**, **(H)** and **(I)**: Gender difference. **(J)**: Representative video tracking images of probe test in the WMT. Values are means ± standard error. *p < .05 was statistically significant.

A repeated MANOVA was used to analyze the mean latency of each group in finding the platform during initial spatial learning with treatment and test days across different genders and separation time of MS. In male rats, the interaction was statistically significant between treatment and time (*F*(9,207) = 10.92, *p* < .001). The results showed that the separate effect that the treatment had was significant from day 1 to day 3 (**Figure 4A**). On day 1, the control groups spent less time finding the platform compared to other groups (PTSD: *p* = .014; MS3h-PTSD: *p* = .008; MS6h-PTSD: *p* = .003). On day 2, the PTSD and MS6h-PTSD groups demonstrated more time compared to the control (*p* = .001; *p* = .007) and MS3h-PTSD (*p* < .001; *p* < .001) groups. On day 3, the MS3h-PTSD group used less time compared to the control (*p* = .003) and PTSD (*p* = .003) groups. However, there were no striking differences on day 4 between any of the treatment groups. In female rats the interaction between treatment and time was statistically significant (*F* (9,207) = 12.73, *p* < .001). Further analysis of the female groups showed that the separate effects of treatment was significant on day 1, day 3 and day 4 (**Figure 4B**). On day 1, the control group used significantly fewer time compared to the rest groups (PTSD: *p* = .002; MS3h-PTSD: *p* < .001; MS6h-PTSD: *p* = .028) apart from that MS6h-PTSD group had significantly less time compared to the PTSD (*p* = .004) and MS3h-PTSD group (*p* = .001). No striking difference was found on day 2 between any of the groups. On day 3, the PTSD group spent less time compared to other groups (Control: *p* = .024; MS3h-PTSD: *p* = .021; MS6h-PTSD: *p < .001), and the MS3h-PTSD group used less time compared to the MS6h-PTSD group (*p* = .006). On day 4, the MS3h-PTSD group used less time compared to the control group (*p* = .001) as well as the MS6h-PTSD group (*p* = .010), and the MS6h-PTSD group used significantly more time compared to the PTSD group (*p* < .001). When we explored rats controlling for gender difference, the interaction was statistically significant between treatment and time (*F*(9,423) = 16.16, *p* < .001). The separate effects of treatment were significant from day 1 to day 4 (**Figure 4C**). On day 1, the control group used less time compared to other groups (PTSD: *p* < .001; MS3h-PTSD: *p* < .001; MS6h-PTSD: *p* < .001). On day 2, the PTSD group used more time compared to the control group (*p* = .005) and the MS3h-PTSD group (*p* < .001). On day 3, the control group required more time compared to the PTSD group (*p* = .017) and the MS3h-PTSD group (*p* = .003), and the MS6h-PTSD group used more time compared to the PTSD group (*p* = .027) and the MS3h-PTSD group (*p* = .005). On day 4, the control and MS6h-PTSD groups used more time respectively compared to the PTSD group (*p* = .045, *p* = .036) and the MS3h-PTSD group (*p* = .001, *p* = .010).

The separate effect that the test day had from day 1 to day 4 was significant in all treatments no matter if the rats were male (Control: *p* = .001; PTSD: *p* = .003; MS3h-PTSD: *p* = .001; MS6h-PTSD: *p* < .001), female (Control: *p* = .003; PTSD: *p* < .001; MS3h-PTSD: *p* < .001; MS6h-PTSD: *p* = .001) or from the group without gender difference (Control: *p* < .001; PTSD: *p* < .001; MS3h-PTSD: *p* < .001; MS6h-PTSD: *p* = .000), suggesting that the groups’ learning ability improved after the treatment test.

The results of the probe test revealed gender differences between rats in the MS3h group and the PTSD group; additionally, the M-MS3h-PTSD rats showed a higher spatial preference for the target quadrant than the F-MS3h-PTSD rats (*F*(5,66) = 2.72, *p* = .034). No significant differences were found between treatments (**Figure 4D**). A higher proportion of M-Control could be seen, compared with the M-PTSD and M-MS6h-PTSD groups, with significant differences (*p* = .014, *p* = .002; **Figure 4D**). When male and female rats were studied together without gender difference in WMT, the control groups required more quadrant time compared with rats in other groups, while there were no significant differences among the experimental groups (**Figure 4E**). The gender difference here is not significant (**Figure 4F, G, H and J**) and the tracking images was shown in the figure (**Figure 4J**). Detailed data information can be found in the **Supplementary Material**.

### Analysis of Depressive Behavior

**Figure 5:**
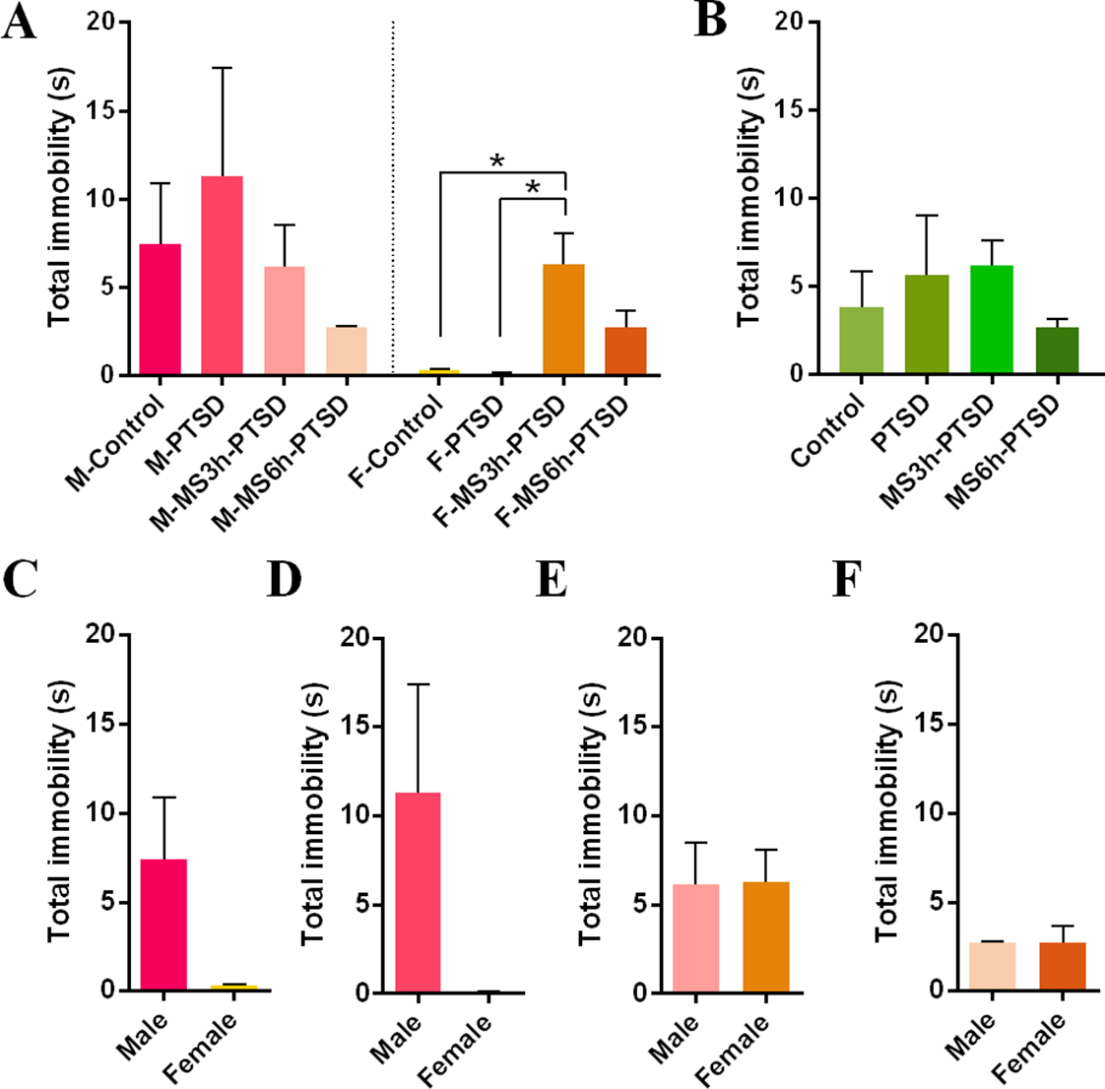
Depression-related effect that MS had from PND 2 to PND 14 after subjecting the animals to the process of SPS as a PTSD model in adult age. **(A)** and **(B)**: Total immobility(s) in the FST. **(A)**: Divided by gender and time difference of MS. **(B)**: Divided by time difference of MS. **(C)**, **(D)**, **(E)**, and **(F)**: Gender difference. Values are means ± standard error. *p < .05 was statistically significant.

The F-MS3h-PTSD group showed more immobility than the F-PTSD group (*F*(3,20) = 8.49, *p* = .001; Post hoc test: *p* = .001) and no immobility difference in male rats (**Figure 5A**). No gender differences were found in terms of immobility between the MS3h-PTSD groups and MS6h-PTSD groups (**Figure 5A**). The F-MS3h-PTSD groups had more immobility time compared to the F-Control rats (*F*(3,20) = 8.49, *p* = .002). No significant differences emerged in depressive-like behavior between the MS3h-PTSD, MS6h-PTSD, and PTSD groups when there was no gender distinction (**Figure 5B**). The gender difference here is not significant (**Figure 5C, D, E and F**). Detailed data information can be found in the **Supplementary Material**.

## Discussion

Several lines of evidence have showed that in stressful contexts under MS conditions, physiological systems prepare themselves more efficiently (11). MS changes can be regarded as adaptive modifications. If animals are raised in stressful environments early, the nervous system, immune systems, and the endocrine system will be altered to cope with stressful conditions later (26, 37). This finding differs from previous theories positing that early adverse experiences represent a risk element for developing anxiety disorders. In this research, we performed MS 3h or MS 6h daily from PND 2 to PND 14 to mimic adverse early life stress along with the SPS process to mimic secondary stress in adulthood, resulting in PTSD.

The impacts of MS on the rats’ neurobehavioral development remain ambiguous as a result of the varying patterns observed by researchers. Some have shown that the impacts of MS depend on separation conditions (e.g., length and time of separation, gender, environmental conditions such as ambient temperature, and genetic background) (38, 39). Care must be taken when implementing MS, as pups are affected by their mothers’ odor or sounds; thus, it is important to ensure complete separation to guarantee the accuracy of results. In this study, pups were removed from the nest and placed into a 30°C incubator. As one source of error, unscheduled husbandry of cages may lead to high levels of ammonia, which cannot be neglected (40). Results cannot be tied directly to a poor early social environment if pups lose body warmth, because temperature might play a role, hence the recommendation for an incubator to avoid hypothermia (41). These observations show that long-term effects can be influenced by distinct MS conditions. The length and time of day when maternal separation occurs carries significant implications for these effects. In this experiment, differences in daily separation time (3h or 6h per day) and gender were studied. Female rats with 6h MS showed less anxiety than those with 3h PTSD. Increased sensitivity of the pituitary gland to exogenous corticotrophin-releasing hormone administration and the impaired negative feedback regulation of the HPA axis in male rats could explain this phenomenon. We can postulate that the previously mentioned adaptations might be useless in non-stressful conditions, which could lead to neuropsychiatric-like disorders or behavioral impairments. This finding could explain inconsistent conclusions. Potential behavioral consequences, as well as the mechanisms underlying this effect, will require future research.

Several animal models of PTSD, such as those involving social stress and predator stress, have been proposed. These animal models fail to demonstrate the most consistent neuroendocrinologic characteristic observed in PTSD patients, namely enhanced inhibition of the HPA axis, although they present behavioral alterations like PTSD (42–44). The SPS model, proposed by Liberzon et al., replicates the specific neuroendocrinologic abnormalities observed in PTSD patients (45), for example, enhanced glucocorticoid negative feedback. A recent ‘dismantling’ study that employed different components of SPS (i.e., two of three stressors) was compared to the effect of full SPS (involving restraint, forced swim, and ether exposure); only those rats that were exposed to the full procedure exhibited deficits in retention of extinction memories—a mechanism which was thought to contribute to an inability to retain new safe memories and prevent trauma recovery (46). Rats exposed to SPS in this study suggested enhanced inhibition of the HPA system. We used this classical animal model to establish severe PTSD-mimicking trauma in adults who had experienced early stress in their childhood.

In the present study, rats that withdrew from the SPS test exhibited a significant reduction in exploratory activity, such as upright posturing during the OFT (47). Anxiety-like behavior and locomotor activity were evaluated *via* EPMT (48). The increased time spent in the closed arms during a 5-min session was indicative of high anxiety-like behaviors (49). The less time the rats spent in open arms, the more anxious and less curious they were. Although the OFT and EPMT can detect the degree of anxiety, the OFT is suited to measuring spontaneous activity, whereas the EPMT is suited to explore animals’ reactions to novelty in new and different environments. Gender differences were found in adult rats that had experienced MS before PTSD: male rats had significantly fewer upright numbers and less time in open arms in the OFT or EPMT. Female rats with MS 3h were more likely to enter the open arms in the EPMT. Male rats with less locomotor activities, which experienced severely adverse early life conditions (MS 6h), were more likely than female rats to exhibit anxious emotions after the second trauma in adulthood. The female rats showed less anxiety-like behavior and were more curious when subjected to severe early stress (MS 6h). Early adverse experience thus affected males more intensely, despite the fact that that compared to men, women have a higher prevalence of anxiety disorders. We thought the contradiction between these consequences might be attributable to species-specific differences, especially when studying sex differences in the susceptibility to early adverse conditions (50). The timing of estrogen in females should also be considered, as it may influence hormones and neurotransmitters. Anxiety-like behavior has been found to be directly related to certain hormone levels. León Rodríguez and Dueňas et al. found that females rats that experienced maternal separation from their mothers (6h per day, PND 1 - 21) displayed decreased levels of anxiety and impulsive behavior and were more active than their stressed male counterparts, consistent with our findings (51). Wang et al. employed repeated MS (one daily period of 4h from PND 1 to 21) and found no significant gender differences in anxiety or locomotor activity in the OFT, regardless of age (52). Some studies suggested that MS could exacerbate the result of exposure to a PTSD model in exploration activities, at least in male animals. Prut et al. discovered anxiety-like behavior in MS male rats, as determined by the time spent in the central area in OFT, which implied aggravation of the anxiety-like degree (i.e., increase of movement time in the surrounding area) when exposed to dangerous environments; however, this impact was not observed in females. Our study revealed no significant differences between the experimental group regarding anxiety as represented by the time entering the central area of the OFT regardless of gender or treatment. We suspect that early life stress might not always contribute to unpredictable traumatic events experienced in adulthood. The OFT material and the different MS operations may have been susceptible to the smell of the rats, which could have influenced the final results.

Spatial learning data from the WMT showed that the M-MS3h-PTSD group had significantly lower mean latency when locating the platform on the second and third test day compared to the rats in the M-MS6h-PTSD group; this result implies that MS for the 3 h/day from PND 2 to 14 slightly strengthened spatial learning in the male adult rats, in line with prior research. This might be because mild early stress was more conducive to spatial learning. A previous study found that 24h maternal separation in PND 9 improved spatial learning (53) but found no significant gender difference in the spatial learning test. Our conclusions differ from those of other studies, and we suspect that this may be due to differences in the design of the experiment, which means that the results of MS are different from the results of PTSD after the MS, although the effects of possible experimental errors need to be taken into account. Our results suggest that the M-MS6h-PTSD group had poor performances in the spatial navigation learning; although the differences did not have statistical significance, the trend is still there. We suspect that 6h of MS could model the impacts of early life stress in cognitive dysfunction during adulthood and might lead to additional general learning deficits later in life. Longer MS could also result in dystrophy, which affects brain size, volume, activity, and the number of brain cells. Research has shown that early adverse experiences resulting from MS 6h could reduce cell proliferation in the dentate gyros of the hippocampus, hinder acquisition of spatial learning (54, 55), lead to neuronal cell death, and eventually cause memory impairment (56). Over time, all male rat groups showed enhanced spatial learning after 4 days’ training in this study, but no significant differences appeared in the rats’ ultimate learning ability in any group. Interactions between an individual genetic profile and the early environment, such as MS, could be involved in adaptive programming, which could amplify responsiveness in animals. Severely adverse early life conditions in female rats (MS6h) affected the spatial learning of the subject, which spent significantly less time on mean latency to find the platform on the first training day and more time on the third and fourth day, suggesting that MS 6 h/day from PND 2 to 14 slightly disrupted spatial learning in adult female rats. A similar situation was found on the third and fourth training days without gender difference. Xiu-Min Sun et al. found behavioral results of the WMT indicating that the rats’ memory and spatial learning ability were impaired by early life stress. Our study found that the spatial probe test showed no significant differences among the MS3h-PTSD, MS6h-PTSD and PTSD groups, implying that rats who experienced trauma early in life and then experienced a second trauma in adulthood do not suffer from effects on spatial memory. Charles V Vorhees and Michael T Williams offered that the number of times an animal floats could be reduced by proper water temperature (57); thus, a more stable temperature would be better during the experimental process.

Immobility is a sign of increased depression-like behavior (49). Female rats that experienced PTSD gave evidence of lower levels of depression when subjected to the FST. This pattern was evidenced by the increased proportion of immobility latencies compared with the F-MS3h-PTSD group, suggesting that female rats that experienced mild early stress (MS 3h) prior to PTSD exhibited more depression-like behavior than the F-PTSD group. These findings were consistent with a study from Hesong Liu, Gaurav Patki et al., which found that experiencing early-life maternal stress causes depression-like behavior later in adulthood for PND 60. This research was found in male and female rats, implying no gender difference in this behavior (58). A difference was found in depression in female rats; differences in experimental design, control group, strain differences, or a combination of these factors might cause such discrepancies, these may also explain the high standard deviation of male rats in the blank control group and the PTSD group.

Our study utilized emotion- and cognition-related behavioral assessments of adult rats following induced post-traumatic stress disorder after various lengths of maternal separation to explore the impact of MS on PTSD as well as the effects of different MS periods. Whether the aforementioned adaptations are useful in non-stressful environments remains unknown, although we will continue to explore this potential effect as well as the underlying mechanisms that may result from relevant treatments.

## Conclusions

This study demonstrated that, with early life stress, female rats that showed less anxiety are more resilient to second stress in adulthood compared with male rats. Although there were signs of reduced anxiety in rats with early MS trauma compared with animals with only PTSD, they were not significant. Apart from that, male rats with slight MS exhibited better spatial learning than those exposed to severe MS. In terms of depression, female rats that experienced mild MS showed no adaptation to trauma but exhibited increased depression-like emotions. Although different results were obtained with different MS intensities, our data suggest that under several circumstances, early life stress could be adaptive in some respects, especially in spatial learning. This feature could play a role in predicting the emotional and cognitive outcomes of left-behind children who experience trauma in adulthood.

## Ethics Statement

Our study followed the conventional requirements of experimental operation and was approved by the ethics committee of Weifang Medical University.

## Author Contributions

LS, RY, HRS and YW designed the study. RY, HRS and YW collected the data. RY analyzed data and drafted the manuscript. GL, YW, QL, JZ, HWS and LS reviewed the manuscript. All authors contributed to and approved the final manuscript.

## Funding

This research was supported by the Youth Natural Science Foundation of Shandong Province (ZR2017QH001), the science and technology foundation program of the colleges and universities of Shandong province (J17KB096), and the research award foundation program for outstanding young scientists of Shandong province (BS2014YY043), and the Students’ Research Fund of Weifang Medical University.

## Conflict of Interest Statement

The authors declare that the research was conducted in the absence of any commercial or financial relationships that could be construed as a potential conflict of interest.
